# A template-free, more environmentally friendly approach for glass micro-texturing

**DOI:** 10.1038/s41598-022-04930-8

**Published:** 2022-01-18

**Authors:** Yuhui Jin, Aize Li, Ross J. Stewart, Robert R. Hancock, David E. Baker, Ruchirej Yongsunthon, Kelleen K. Hughes, David L. Weidman

**Affiliations:** 1 Corning Research and Development Corporation, Corning, USA; 2 Corning Inc., Corning, USA

**Keywords:** Synthesis and processing, Inorganic chemistry, Materials chemistry, Surface chemistry

## Abstract

Micron and nanometer size textured silicate glass surfaces are of interest in consumer electronics, photovoltaics, and biosensing applications. Typically, texturing glass surfaces requires applying a patterned mask or a pre-etching treatment (e.g. sandblasting) on the glass substrate, followed by a mask transferring or etching process using a fluoride-containing compound. The major challenges of such a process are the complexity and cost of masking, and the safety and environmental concerns around the usage and disposal of hydrofluoric acid. Here, we describe a template-free method to construct micron-sized and submicron-sized texture on isotropic glass surfaces in one step. The new texturing mechanisms are well supported by experimental data and peridynamic simulations. With this novel strategy, the etchant uses fluoride-free chemicals such as citric acid to texture silicate glass. Etchant concentration, etch temperature, time, and additives are the primary parameters that dictate the texturing process. Surface feature size and depth can be independently controlled by tuning the leaching and chemical polishing process. We hope this study can trigger more research on novel and more environmentally friendly texturing of isotropic materials.

## Introduction

Microtextures play critical roles in defining material surface functions and properties^1–5^. In nature, surface microtextures allow living creatures to develop critical functions and skills. For example, lotus leaves with micron-sized wax features exhibit ultra-hydrophobicity and self-cleaning properties^1^. Moth eyes with submicron-sized pillar arrays possess an antireflection function to reduce light reflection^2,6^. Previous works have recreated some of these natural occurrences through surface engineering. The engineered microtextured surfaces show desirable functions such as anti-reflection, antiglare, hydrophobicity, and haptic feedback^3–5,7–9^. Glasses with engineered microtextures are of great interest in consumer electronic markets^10^, and biosensing^8^, photovoltaic applications^4–6,11^.

Silicate glass surfaces are typically textured by sandblasting and etching, wet chemical etching, and sol–gel deposition processes^12–15^. In these processes, random surface features of ten to hundreds of microns in size are generated by mechanical subsurface damage followed by etching^13^, in-situ growth of inorganic micro-structural masking on glass during etching^12,14^, or the non-conformal deposition of silica sol–gel droplets on a glass surface^15^. Nanolithography and a few other mask and etch approaches are employed to generate smaller features ranging from submicrometer to micrometer on the surface^5,9,16–18^. However, the masking process is expensive and infeasible for large scale manufacturing. In addition, the use of hydrofluoric acid (HF) or fluoride compounds in the above-mentioned techniques for microfabrication, raises concerns for safety and waste disposal^19,20^.

## Results and discussion

### Mechanism

**Figure 1:**
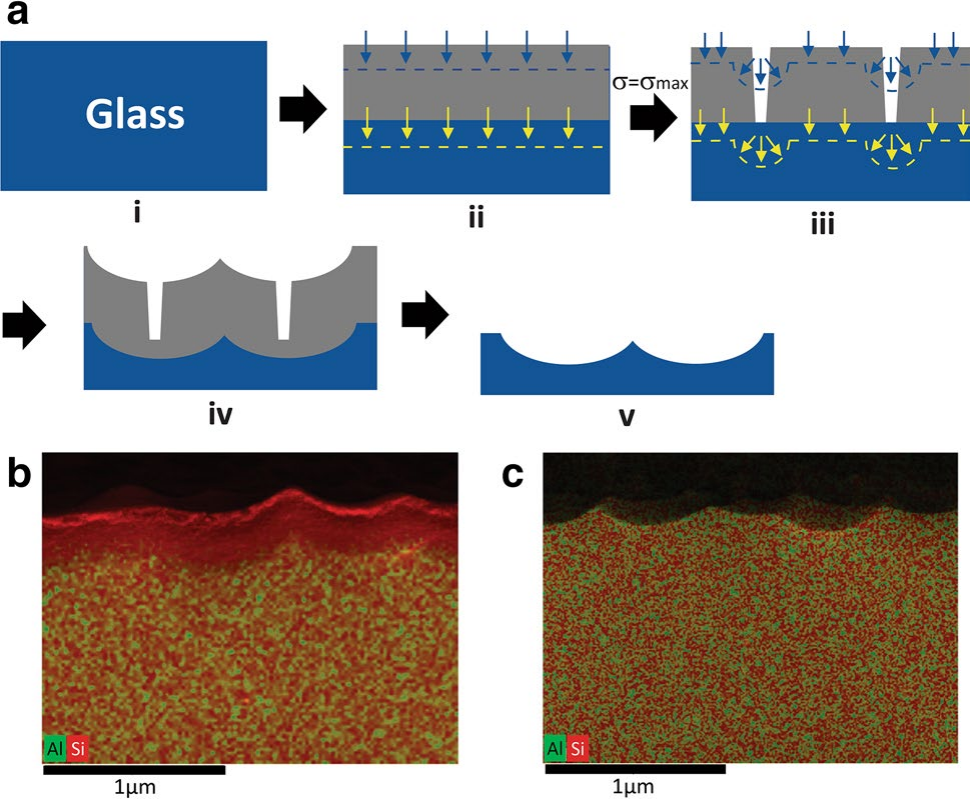
Mechanism of differential leaching and texturing of silicate glass. (**a**) Process flow and glass surface evolution through differential leaching and texturing. Structure i represents a 2D cross-section of a silicate glass. During leaching, the glass surface starts to develop a uniform silica leaching layer marked in grey color in structure ii. The yellow arrows indicate the direction of uniform leaching. The blue arrows are the direction of uniform silicate dissolution. The grey leaching contains a surface stress value (σ). When surface stress (σ) equals to the critical pore formation stress (σ_max_), critical pores are generated through the leach layer as shown in Structure iii. The formation of the critical pores initiates the differential leaching and silica dissolution. The directions of differential leaching and silica dissolution are indicated by the yellow and blue arrows in structure iii, respectively. In this step, textures are developed at both leach layer surface and the bulk glass beneath the leach layer (structure iv). The leach layer can be removed by a high-pH treatment, which reveals the textured glass surface (structure v). (**b**) SEM/EDS image of cross-section of an aluminosilicate glass (Glass A) after differential leaching in 20 wt% citric acid at 95 °C for 10 h. The scale bar of this image is 1 micron. In the EDS map, Si elements are marked in red and Al elements in green. The top surface shows a textured silica leaching layer of about 300 nm. The bulk glass (with both Si and Al) beneath the leaching layer also possesses textures. The glass surface structure is the same as structure iv described in process a. (**c**) The textured glass (characterized in image (**c**)) was further washed in pH 12 NaOH solution at 60 °C for 2 min to remove the leaching layer. The cross-section of the revealed textured surface was characterized by SEM/EDS (with 1-micron scale bar). The uniform distribution of Al and Si elements indicates the leaching layer is completely removed during the washing step.

To overcome these challenges, we developed a low pH differential leaching process without HF to texture silicate glass surfaces (Fig. 1, Process a)^21^. Glass leaching is a process through which glass modifiers and some glass formers can be exchanged from the silica network by hydronium ions (H_3_O^+^) under acidic conditions^22,23^. At the initial stage of glass leaching, a uniform silica enriched leaching layer is formed on the glass surface (Fig. 1, structure ii). The leaching layer exhibits low surface roughness and no apparent optical haze. Because the leaching layer has a lower molar volume compared to the bulk glass and tends to shrink, the thickening of the leaching layer starts to build up a surface tensile stress (σ). Gradually, silica moieties from the leaching layer also start to dissolve in aqueous media which slows down the leaching layer growth. For glasses with high silica content or low leachable materials, a shallow and uniform leaching layer is usually formed and maintained as the leaching and silica dissolution processes reach equilibrium with constant leach layer thickness (Fig. 1, structure ii). In this scenario, both the direction of leaching and silica dissolution (as indicated by the blue arrows and yellow arrows in Fig. 1a, structure ii) are uniformly aligned and perpendicular to glass surface. For glasses with enough leachable materials, both leaching layer thickness and surface tension (σ) continue increasing until the tension exceeds the critical stress for effective pore formation (σ_max_). The high tensile stress leads to cracking of the leaching layer and the development of numerous deep pores (Fig. 1, structure iii). These effective pores modify the localized diffusion rate and promote an uneven growth of the leaching layer and preferential silica dissolution around the pore location. Structure iii in Fig. 1a illustrate the direction of leaching (yellow arrows) and silica dissolution (blue arrows) are changed and accelerated at the pore location. As a result, a micro-textured topography forms both at the leaching layer surface and at the leaching-bulk glass interface (Fig. 1 structure iv). After removing the porous leaching layer at a high pH condition, the textured glass surface is revealed (Fig. 1, structure v). Overall, the differential leaching process can be divided into three stages. Stage One is the growth of the uniform leaching layer where the surface exhibits low roughness without apparent haze. The second stage begins as the critical tension (σ_max_) is reached. The formation of critical pores sets off the differential leaching and texturing. In this stage, haze grows rapidly until it reaches saturation (transmittance haze can be up to 100%). In the third stage, surface texture and haze reach a steady state. It is worth noting that this differential leaching and texturing process only happens on glasses with an appropriate leaching rate. Slow leaching cannot generate enough tension to create critical pores in the leaching layer. Rapid leaching on a highly leachable glass often generates enormous surface tension which causes surface delamination instead of texturing^23^.

In this study, we demonstrate the differential leaching and texturing process by texturing an aluminosilicate glass with 63.6 mol% of SiO_2_ (Glass A) in 20 wt% aqueous citric acid solution at 95 °C for 10 h. Through this condition, a 300 nm-thick uniform leaching layer is quickly formed on the glass surface in the first 5 h of the leaching process. The follow-up differential leaching process creates textures on the silica-enriched leaching layer due to the differential silica dissolution at the critical pore locations (Fig. 1b, and supplementary Fig. S1). The cross section of the leached glass clearly shows the silica enriched leaching layer (Si in red) in comparison to the bulk which contains both Si (red) and Al (green) elements characterized by Scanning Electron Microscopy (SEM)/ Energy Dispersive X-Ray Spectroscopy (EDS). In the leaching layer, alumina and other components are leached out and leave a porous network with 97 mol% or more SiO_2_ characterized by Second Ion Mass Spectroscopy (SIMS) (Supplementary Fig. S1). Interestingly, the differential leaching also sculpts a textured interface between the silica leaching layer and the bulk glass. Because both differential silica dissolution and differential leaching are concurrently triggered and developed at critical pore locations, the associated texture profiles are almost parallel to each other and share great similarities in terms of geometry and feature sizes. The porous silica leaching layer is removed in high pH solution (pH > 14), the bulk textured surface is revealed, as shown by the cross section of the SEM/EDS image (Fig. 1c). In the following experiment, all glasses are first treated in acid to generate surface texture, and then briefly washed by high pH detergent to reveal the bulk textured surface.

### Main factors (etchant concentration, etch time and temperature)

**Figure 2:**
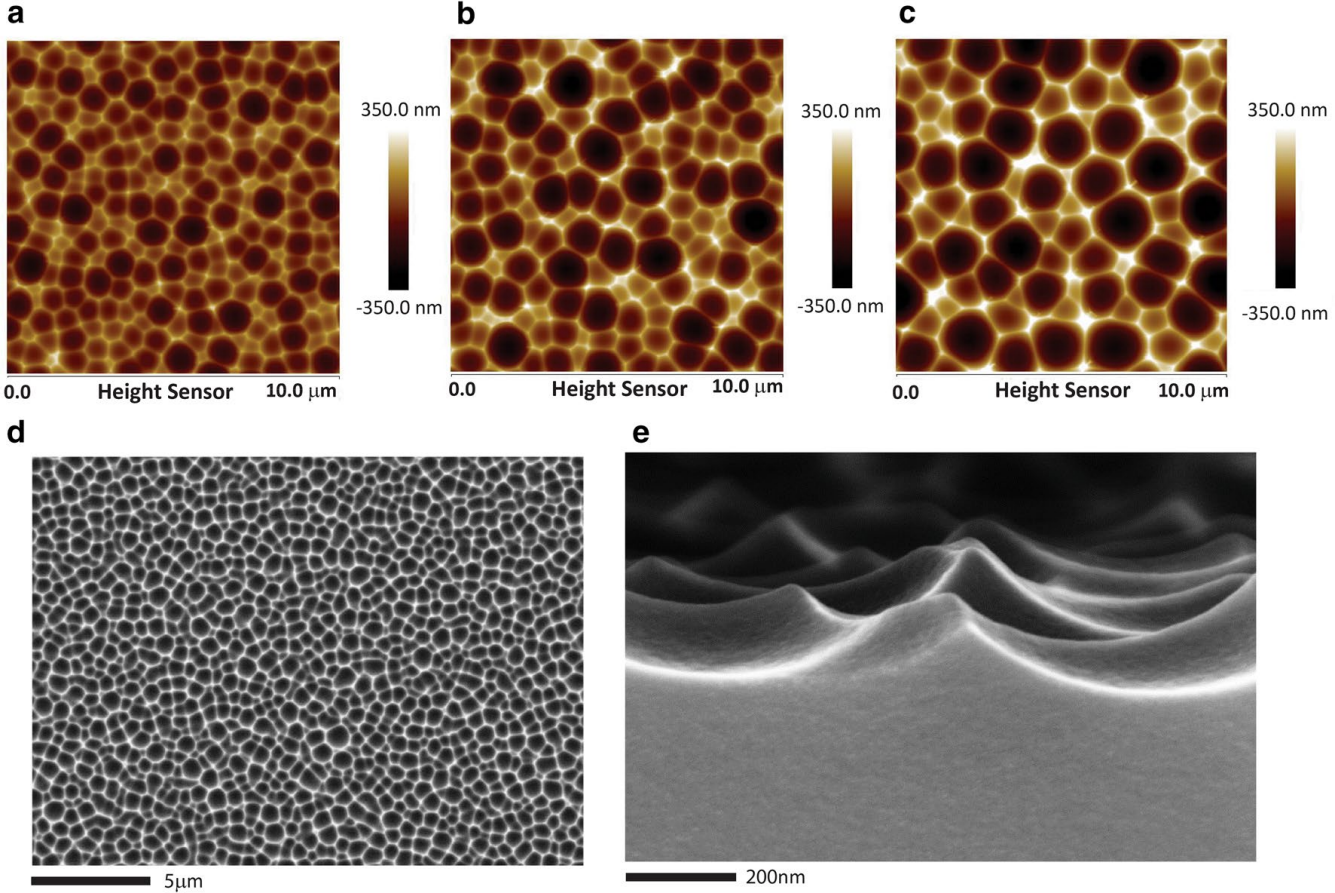
SEM and AFM images of textured aluminosilicate glass surfaces (Glass A) after citric acid and NaOH treatments. (**a**)–(**c**) 10 µm x10µm AFM images of textured Glass A treated in 20 wt% citric acid at 95 °C and a follow-up NaOH washing. The image color scale is mapped to quantitative height data. The citric acid treatment times for images (**a**)–(**c**) are 10, 13.5, and 16 h, respectively. The textures on (**a**)–(**c**) possess continuous concave features with diameters of 0.8, 1.2, and 1.5 microns, and surface roughness (Ra) values of 65.7, 94.8, and 120.3 nm. These textured glasses (with one-side texture) contribute transmittance haze values of 19.5%, 38.8%, and 59.0%. Longer acid treatment time leads to larger surface features, higher surface roughness (Ra), and higher haze values. (**d**) SEM image of the textured surface also shown in image (**a**). The scale bar is 2 microns at 5kx magnification. A uniform texture surface is observed. (**e**) SEM cross-section view of the 10-h treated surface [same surface of image (**a**,**d**)]. The scale bar is 200 nm at 100kx magnification. As shown by the SEM images, the textured surface consists of pointed peaks connected by curved ridges, and concave valleys surrounded by the peaks and ridges.

**Figure 3:**
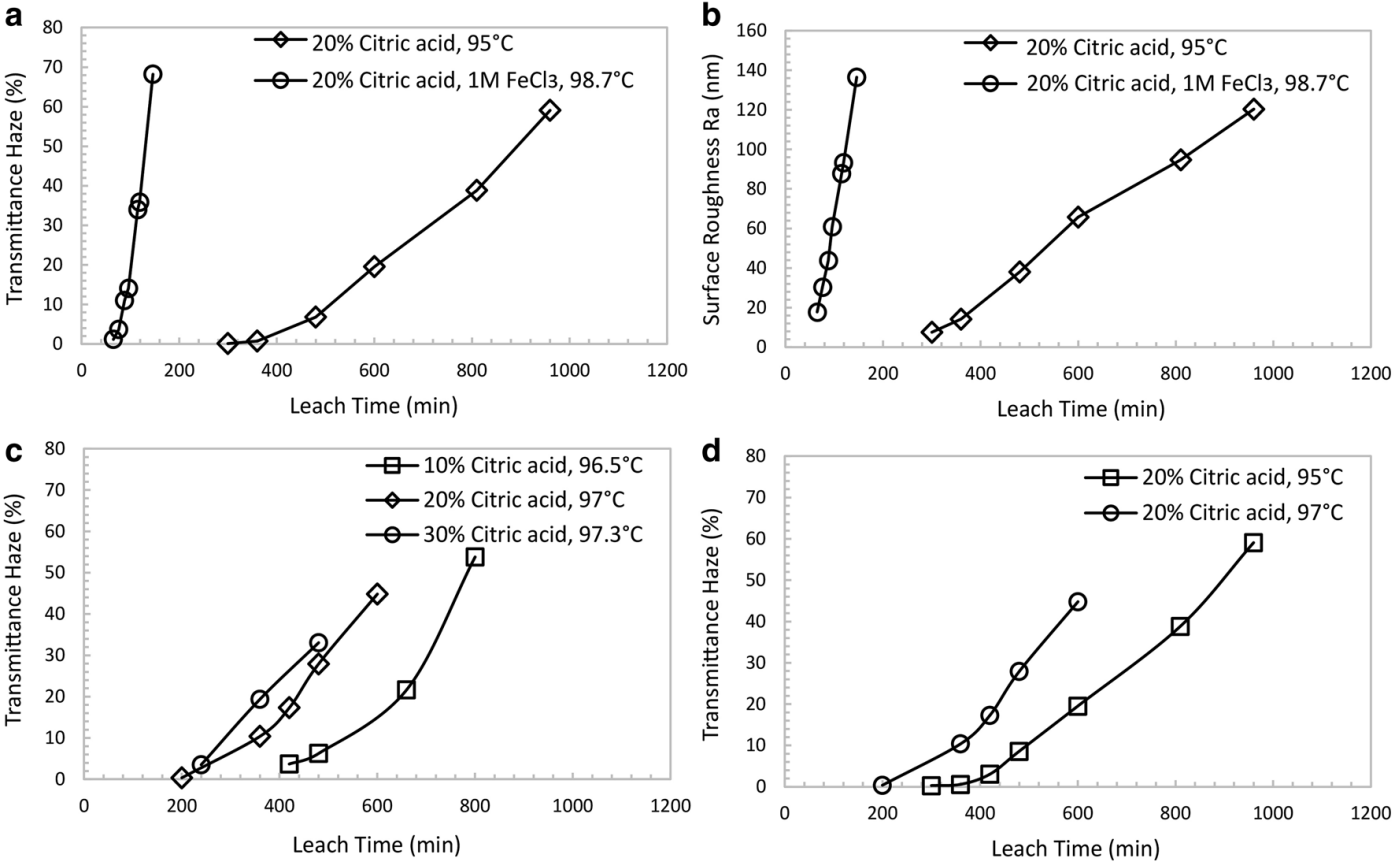
Process factors influencing differential leaching and texturing process using Glass A as examples. All glass articles were first treated in acid solutions and then washed in NaOH solution to reveal the textured glass surface. Surface roughness (Ra) was measured by AFM. Single-side transmittance haze values were measured by BYK Haze-Gard. (**a**), (**b**) The impact of leach time on surface haze development (**a**) and surface roughness development (**b**). Plots with diamond markers (◊) are the haze values (**a**) and roughness values (**b**) of textured Glass A treated in 20 wt% citric acid at 95 °C for different leach time. Plots with circle markers (○) present the haze development (**a**) and roughness development (**b**) of Glass A in 20 wt% citric acid and 1 M FeCl_3_ at 98.7 °C for different leach time. Both plots confirm the differential leaching process contains a uniform leaching stage without haze (Stage I in Fig. 1 diagram b) and a differential leaching stage (stage II in Fig. 1 diagram b) with rapid haze growth. The usage of FeCl_3_ significantly accelerates the texturing process. **c**, The impact of citric acid concentration on haze development. Glasses were textured in 10 wt% (labelled as square markers: □), 20 wt% (labelled as diamond markers: ◊), and 30 wt% (labelled as circle markers: ○) citric acid solutions at 96.5 °C, 97 °C, and 97.3 °C, respectively. Glass texturing process is shortened by increasing citric acid concentration. **d**, The impact of leach temperature on haze development. Glasses were textured in 20 wt% citric acid at 95 °C (square markers: □) and 97 °C (circle markers: ○). Data show that increasing treatment temperature promotes a quicker texturing process.

The texturing process is controlled by a few factors including acid solution concentration, leaching time, and leaching temperature. As depicted by Fig. 1a, a uniform leaching layer is formed in 20 wt% citric acid at 95 °C, and continuously grown in the first 5 h. As the critical pores are formed in the leaching layer, surface texture begins to grow from the fifth hour. After 10, 13.5, 16 h of the acid treatment and a consecutive high pH cleaning, the glass A surface shows continuous concave features of 0.8, 1.2, and 1.5 microns in diameter as characterized by atomic force microscopy (AFM) and SEM (Fig. 2a–e). Longer differential leaching time results in larger and deeper surface features or higher surface roughness (Ra), and the higher surface roughness leads to higher transmittance haze. Both the surface texture roughness (Ra) and surface feature size are linearly correlated with the second stage (structure iii, Fig. 1a) treatment time (Fig. 3b).

The glass leaching process is highly influenced by leaching solution pH (acid concentration) and temperature^22^, as is the differential leaching and texturing process. Figure 3c and d show that the Glass A surface exhibits higher haze when the treatment temperature is changed from 95 to 97 °C (Fig. 3d). Increasing citric acid concentration within certain range also accelerates the texturing process. Increasing citric acid concentration from 10 to 20 wt% significantly reduces texturing time for Glass A. While, further elevating the citric acid concentration from 20 to 30 wt% does improve the texturing rate (Fig. 3c). Besides citric acid, other weak acids such as malic acid, lactic acid, and tartaric acid, can also texture Glass A due to their similarities in acidities (pKa) (Supplementary Table S1). Interestingly, when strong acid (such as hydrochloric acid, HCl) is used to treat Glass A, the lower pH solution treatment (with much higher hydronium ion concentration) does not texture the surface of Glass A but causes surface delamination. The severe leaching process leads to uncontrollable stress build-up and far exceeds the pore-formation critical tension (σ_max_), which delaminates the Glass A surface.

### Glass composition

Glass composition, especially SiO_2_ concentration, dominates the glass leaching behavior, and is another key factor that determines the texturing process. For example, a more chemically durable glass (Glass B with 67.5 mol% SiO_2_ content) is textured in 5 wt% HCl solution at 95 °C, but not in citric acid solutions of any concentrations, while, Glass C with 70.9 mol% SiO_2_ shows no surface texture even after being treated with strong acid. In general, the differential leaching and texturing process works for the tested glasses containing 63.6–67.5 mol% SiO_2_ when an appropriate and controllable leaching process is implemented (Supplementary Table S1).

### Process accelerated by additives

One challenge of differential leaching and texturing is that the process is relatively slow due to the long leaching time before the initial texture formation. To further accelerate the glass leaching, multivalent metal ions (such as Fe^3+^, Al^3+^, Ca^2+^, Mg^2+^ ions) are added to the leach solution. Multivalent metal ions can effectively precipitate soluble silicate ions in the leach solution and accelerate the glass leaching process^24,25^. In this study, the addition of 1 mol/L of FeCl_3_ to 20 wt% citric acid effectively reduces the stage I time from 5 h to about 65 min (Fig. 3a). Figure 3b shows that metal ions with higher ionic charges are more effective in leaching acceleration (Supplementary Table S1). Monovalent metal ions (e.g. Na^+^ ions) slow down the leaching process of Glass A because these ions cannot effectively remove dissolved silicate from the solution.

### Tuning feature size by NaOH polishing

**Figure 4:**
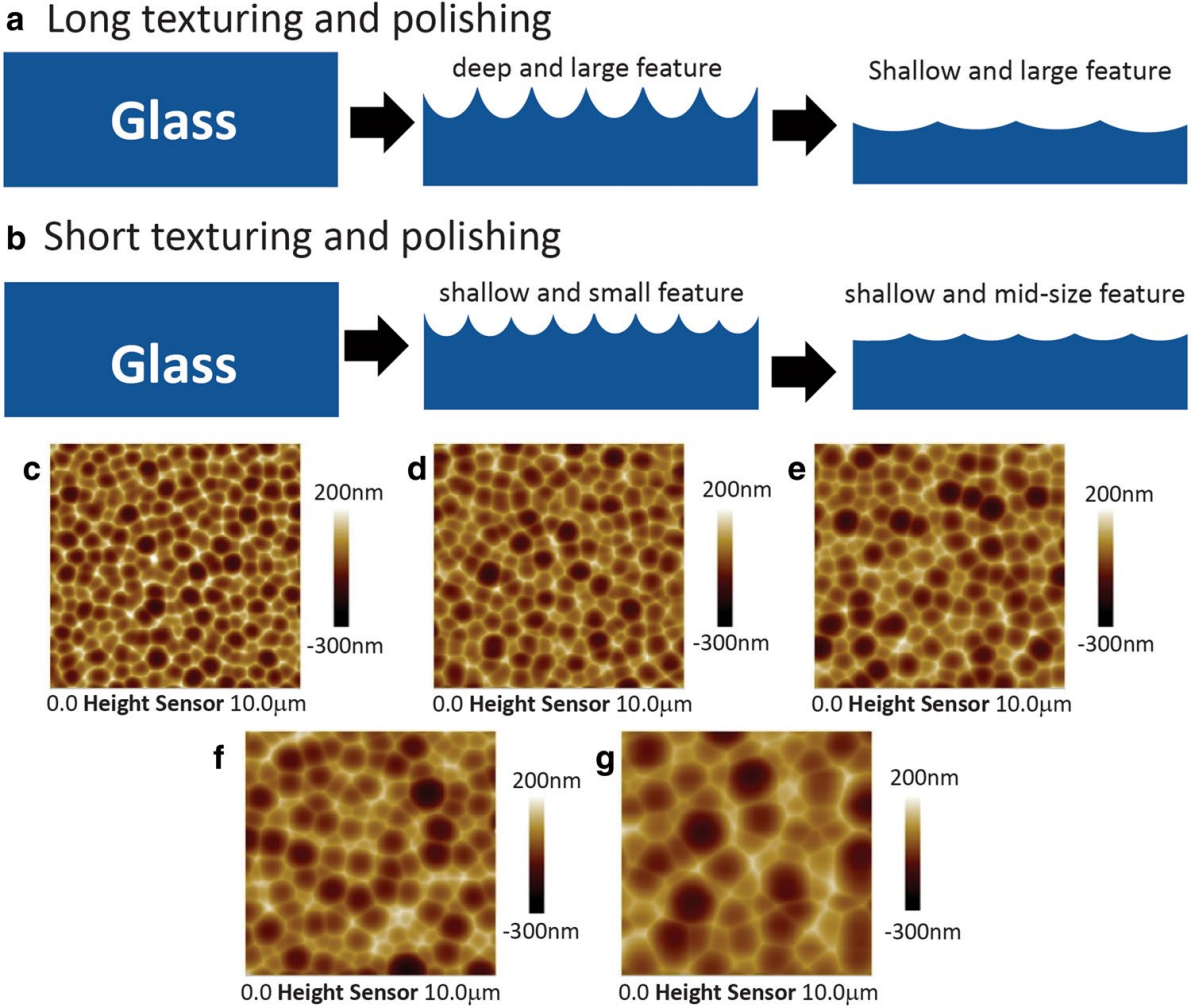
Mechanism of texturing and chemical polishing of silicate glass to tune surface feature size and depth. In this process, the texturing step increases both surface size and feature depth or roughness. The following polishing step grows feature size but reduces texture depth or roughness. (**a**) Generate shallow and large features on glass by long texturing and long chemically polishing. During the texturing process, long treatment results in deep and large features. Prolonged chemical polishing creates shallow and large feature. (**b**) Generate shallow and mid-size features on glass by short texturing and short chemically polishing. During the texturing process, short treatment results in shallow and small features. Brief chemical polishing keeps the surface features small and shallow. (**c**)–(**g**) Examples of texture surfaces at haze level but with different surface feature sizes fabricated through texturing and chemical polishing process (Condition A1–A5 in Table 1). By gradually increasing the leaching time and polishing time, the average feature size increases significantly from 0.9 to 2.3 µm.

As depicted in Fig. 2, both surface texture size, depth, and transmittance haze grow simultaneously during the leaching process. Texture surfaces of different haze levels can be achieved by tuning leaching time, temperature, or etchant concentration. However, the leaching process itself cannot independently adjust surface feature size and depth if the target haze level is fixed. This challenge is solved by adding a chemical polishing step after the acid leaching process. In this two-step process, surface features first grow large and deep and haze increases during the acid leaching step, and then surface features grow larger and shallower and haze decreases during the chemical polishing step (Fig. 4a,b). Conceivably, textures at the same haze level but with different feature size and depth ratio can be achieved by choosing a long leaching and polishing path (Fig. 4a) or choosing a short leaching and polishing path (Fig. 4b). Using this strategy, we fabricate texture surfaces of various aspect ratios at 10% transmittance haze level (Condition A1–A5, Table 1). These surfaces possess similar surface roughness (Ra) of around 40 nm, and average feature diameters (d) from 0.9 to 2.3 µm (Fig. 4c–g, and Table 1). From the process perspectives, the large texture features are achieved by a prolonged leaching process to get haze much higher than the target haze (10% in this case), and then using 10 wt% NaOH to chemically polish the surface and reduce the haze to the target value. In this example, the aspect ratio (d/Ra, nm/nm) is tuned between 18 and 55 (Table 1).

**Table 1:** Adjust texture feature aspect ratio by leaching and chemically polishing.

Condition	leaching time (min)	Polishing time (min)	Post-leaching haze (%)	Post-polishing haze (%)	Post- polishing Ra (nm)	Post-polishing average feature size (µm)	Post-polishing aspect ratio (Feature size/Ra)
A1	94	0	10.4	10.4	48.0	0.9	18
A2	99	2	14.1	10.8	42.4	1.0	24
A3	104	7	17.2	11.0	45.8	1.2	26
A4	110	23	22.3	10.4	43.6	1.5	34
A5	120	63	31.2	10.8	42.1	2.3	55

Leaching is conducted in 20 wt% citric acid and 1 M FeCl_3_ at 98.7 °C. Post-leaching chemically polishing is conducted in 10 wt% NaOH at 95 °C.

### Modeling

To further validate the differential leaching and texturing mechanism, a modeling study was conducted on the continuum level combining mechanical, diffusion, and pore formation methodologies (which are required to model the processes in Fig. 1a, from stage i to stage v). This model utilizes the peridynamic method as the main framework, due to its natural ability to capture elastic material responses, damage and crack formation^26^. This method has been previously used to model corrosion type problems^27^. In this work the diffusion capability is adapted to model both the leaching and chemical polishing processes individually with their respective parameters.

The first step is to model the stress buildup during the initial leaching process (From stage i to stage ii in Fig. 1a). Fick’s second Law (Eq. 1) is overlain on the model to simulate the diffusion process^28^. Where *D* (Eq. 3) represents the location, concentration, and void fraction dependent diffusion coefficient. The parameter *φ* is the location specific leachant concentration (hydronium in the case of acid leaching). The leaching introduces tensile stress (*σ*) calculated by Eq. (2) with constant *C* as a scaling factor, analogously to thermal expansion.

1 $$ \frac{{\partial \varphi \left( {x,t} \right)}}{\partial t} = \nabla \cdot \left( {D\left( {x,\varphi ,v} \right)\nabla \varphi \left( {x,t} \right)} \right) $$

2 $$ \sigma \left( {x,t} \right) = C\varphi \left( {x,t} \right) $$

**Figure 5:**
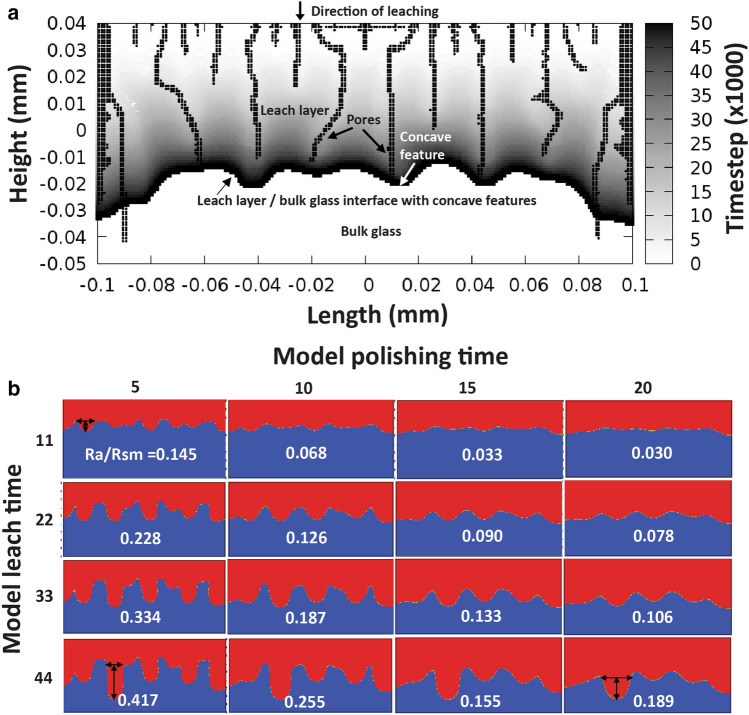
Modeling differential leaching and texturing process. (**a**) An “easily” hydrolyzed model with low value for the network strength (1 J/m^2^), and “wide” pore channels having greater pore diffusivity than the leach layer’s (F_pore_ = 30). The contours represent a growing formation of a textured interface between the leaching layer and bulk glass with the simulation time step. The small black dots represent the effective pores via material points with void fractions greater than 0.2. The effective pores (penetrating the leaching layer depth) create concave features at the interface, due to higher diffusivity within pores. The cross-section resembles the late stage iii shown in Fig. 1a. (**b**) Textured surfaces created by modeling etching and chemical polishing processes. Textures in the same row are modeled with constant leach time, textures in the same column are modeled with constant polishing time. Surface texture shape is defined by surface roughness (Ra, indicated by vertical arrows), feature size (Rsm, indicated by the horizontal arrows), and aspect ratio (Ra/Rsm). The aspect ratio of each modeled surface is labelled in each texture images. By comparing textures within the same column, surface features grow predominantly deeper and a little wider with longer leach time. By comparing the textures within the same row, surface features become shallower and wider.

The second step is to model the pore formation and the start of differential diffusivity (Stage iii in Fig. 1a). When the built-up tensile stress (σ) exceeds the critical stress of pore formation (σ_max_), pores will generate perpendicular to the leaching layer (Fig. 1a, Stage iii). The model achieves this damage by removing material interactions, causing local stresses to redistribute, causing further pore growth beneath existing pores, creating a pore channel (seen in Fig. 5a).The formation of the pore further increases the diffusion coefficient in the leach layer described by Eq. (3). In this equation, ʋ is the void fraction, and *F*_*pore*_ is the diffusivity increase due to the void fraction.

The diffusivity of the leach layer is also assumed to have a linear dependence on the acid concentration, such that higher acid concentration indicates a less dense structure with higher diffusivity. Equation (4) expresses a possible linear dependence when the leach layer is fully devoid of ions and fully saturated with acid, its diffusivity would be, $F_{g,l}$, times as diffusive as the bulk glass.

3 $$ D\left( {x,\varphi ,v} \right) = D\left( {x,\varphi } \right)_{leach} \left( {vF_{pore} + 1} \right) $$

4 $$ D\left( {x,\varphi } \right)_{leach} = D_{glass} \left( {\frac{{\left( {F_{g,l} - 1} \right)}}{{\varphi_{max} - \varphi_{min} }}\left( {\varphi \left( {x,t} \right) - \varphi_{min} } \right) + 1} \right) $$

Figure 5a shows an example 2D cross-section of the leach layer generated through modeling the differential leaching process. As the leach layer (darker contoured grey area in Fig. 5a) grows, effective pores form and penetrate the depth of the leaching layer. The effective pores instigate the change from uniform leaching to differential leaching, due to the higher diffusivity within pores, creating a leach layer and bulk glass interface containing concave features. The texturing stage modeled in Fig. 5a resembles the texturing stage iii described in Fig. 1a.

The third step of this modeling study takes a leached structure as input and models the chemical polishing process with a diffusion model. It assumes that the polishing solution instantly covers the surface and travels down the effective pore channels. With polishing time, the solution eats away at the leach layer, forming the final texture resembling the cratered surface seen in Fig. 2. The results of the model as a function of leach time and polishing time are seen in Fig. 5b.

The aspect ratio of the surface texture is defined by Ra/Rsm, where Ra is the surface roughness, and Rsm is the average feature size. The model suggests that the leaching process tends to increase surface feature depth and width simultaneously. With fixed polishing time, longer leach time leads to deeper and larger features with higher aspect ratio (by comparing the model generated surfaces in the same columns in Fig. 5b). On the other hand, this model shows that the chemical polishing process further evolves the surface texture to be shallower and larger. For example, with fixed leach time, extended polish time creates shallower and larger features with lower aspect ratio (by comparing the model generated surfaces in the same rows in Fig. 5b). Interestingly, similar feature aspect ratios (Ra/Rsm) are achieved in this model by keeping a constant ratio of leach time and polish time (e.g. model generated surfaces from top left to bottom right diagonally, in Fig. 5b), however this is only due to the particular diffusivity parameters chosen for leaching vs. polishing. These surfaces possess similar feature aspect ratio and distinct Ra and Rsm values. In Fig. 5b, the top left surface feature has shallow depth and small size, while the bottom left surface feature is deeper and larger. Overall, the model confirms that the two-step process (leaching and chemical polishing) can create the texture surfaces with different aspect ratio, surface roughness, and feature size as the experimental data demonstrated (Fig. 4).

## Conclusion

We discovered a new strategy to sculpt submicrometer and micrometer-size textures on silicate glass surfaces through a differential leaching process in acidic solutions. This process does not use chemicals containing HF or fluoride which provides technical solutions to resolve the safety and environment concerns in glass texturing industry. Through the process of texturing and chemical polishing, we can independently tune the surface texture size (average feature diameter) from 0.9 to 2.3 um, and surface roughness (Ra) from tens of nanometers to over hundred nanometers. The experiment results suggest that the texturing process is well controlled by these key factors. First, the appropriate texturing process is mostly effective to glasses with SiO_2_ content from 63.6 to 67.5 mol% and in the etchant to promote differential leaching. Second, higher acid concentration, elevated treatment temperature and time usually benefit a quicker texturing process. Third, the addition of multivalent metal ions can further reduce the texturing time within 2 h. This is because the glass leaching and dissolution is accelerated by removing the soluble silicate ions through the coagulation of silicate and metal ions. In this article, we also provided a plausible glass texturing mechanism which includes three stages: the initial uniform leaching layer formation, differential leaching and texturing, and the steady state with saturated haze and maximized roughness. The mechanism is well supported by both experimental data and an exploratory modeling study. We hope this new texturing strategy provides a more environmentally friendly, cost-competitive path for glass texturing research and manufacturing.

## Methods

### Materials

The three silicate glasses (Glass A, B, and C) in this paper were from Corning® Gorilla® Glass families with silica content of 63.76, 67.37, and 70.9 mol%, respectively. Glass A and B are denoted as Glass 4 and Glass 1 in US Patent 20180282201A1^21^. Glass C is denoted as Ex. H in US Patent US10259746B2 listed in Supplementary Table S1^29^. ACS reagent grade chemicals were purchased from Fisher Scientific Inc. including citric acid anhydrous, malic acid, lactic acid, tartaric acid, 37% hydrochloric acid, and Iron(III) chloride hexahydrate, aluminum chloride, as well as other alkali metal chlorides, and alkaline earth metal chlorides evaluated in this work. Sodium hydroxide (NaOH) of 50 wt% solution was acquired from Fisher Scientific Inc. and further diluted to 10 wt% for leaching layer removal. DI water of 15 MΩ cm^−1^ was employed for solution preparation, glass rinsing and cleaning.

### Equipment

Customized Hotblock was purchased from Environmental Express Inc. to precisely control the leaching temperature. Customized Teflon containers of 2000 mL were ordered from Applied Plastics Technology. A glass condenser was employed to minimize solution volume loss from evaporation in the high temperature leaching process.

### Texturing process

In general, three pieces of 50 mm * 50 mm * 0.8 mm Glass A were held in a Teflon holder and placed in a Teflon container with 1000 mL of fresh prepared 10–30 wt% citric acid solution at 95–100 °C. Under a mild refluxing, the glass articles were treated for a certain time. The treated glasses were rinsed in DI water at 20 °C, washed in a 10 wt% NaOH solution at 60 °C for 2 min to remove the leach layer, and rinsed in DI water at 20 °C, sequentially. The textured glasses were dried in air at room temperature or in oven at 110 °C before characterization. Through the same process procedure, various process parameters were evaluated for glass texturing including acid type and concentration, additive species (e.g. iron(III) chloride) and concentration, and leach treatment temperature and time. For differential glass compositions, different acids were applied in attempt to achieve submicron and micron texture on the glass surface. For instance, citric acid was employed for Glass A texturing, hydrochloride acid was elected for Glass B and Glass C. No texture was generated on Glass C surface due to the high acid durability of the glass.

### Chemical polishing

The Chemical polishing process is utilized in a two-step texturing process to independently tune texture feature size (d) and surface roughness (Ra). Glass is first textured in the acid leaching step as described previously. Then the textured glass is chemically polished or etched in 10 wt% NaOH at 95 °C for a certain time to achieve the target haze level.

### Characterization

Scanning electron microscope (SEM) and Energy Dispersive X-Ray Spectroscopy (EDS) were used to evaluate surface morphologies and acquire compositional information of the textured glass at different stages. Atomic force microscopy (AFM), with a Bruker Dimension FastScan and FastScan-B probes in tapping mode, was used to characterize the textured surface morphology, average feature size, and determine roughness metrics. BYK Haze-Gard I was used to measure transmittance haze according to ASTM D1003 protocol. The transmittance haze value was directly measured for single-side textured glasses. If both sides of the glasses were textured by differential leaching, one of the two textured surfaces was mechanically polished (using CeO_2_ particles to remove 20 microns of the surface). The transmittance haze was measured afterward. Alternatively, transmittance haze (value y%) was measured on two-side textured glass. Then single-side transmittance haze (value x%) is estimated by the Equation: y = 2x − x^2^/100.

### Modeling

The bond-based formulation of the peridynamic method was used with a coupled transient diffusion model, an analogue to thermal conduction and expansion. A critical bond strain was used to nucleate pores that correlates to the explained σ_max_ parameter. The void fraction is calculated based on the fraction of material bonds that have broken for each material point. The upper surface of the 2D model had a fixed concentration boundary condition of 1.0. The body of the glass begins with a zero concentration. Since most of the parameters in this model are relatively unknown, normalized units were used and the emphasis placed on their relative magnitudes. The model parameters used for the simulations are as follows, the Young’s Modulus of the glass is chosen to be E = 72 GPa, the fracture energy, related to σ_*max*_, of the leach layer was parameterized and ranged from G = 1–12 J/m^2^. The density of the glass is set to 2400 kg/m^3^ which is only used for the structural-mechanical dynamic response. The 2D glass model prescribes a constant out-of-plane zero strain. The concentration to strain relation is $C = - \,0.001/E$, the acid concentration capacity set to 1.0 and diffusivity for the glass, $D_{glass} = 10$, the interface of glass-acid solution as 200, and within the solution as $D_{acid} = 400$. For the acid leaching step, (Eq. 3) the concentration diffusivity dependence on the pore fraction, $F_{pore}$, ranges from 5 to 30 and concentration, $\varphi_{min}$ is set to be 0.05 and $\varphi_{max}$ to be 1 and $F_{g,l}$ = 10. For the chemical polishing step, the same concentration dependent diffusivity is used for the leach layer, but the initial concentration is set to 1.0 at the upper surface and along all effective pore channels and 0.0 everywhere else. This means that in a fully polished region the diffusivity would be $10D_{glass} = 100$.

The geometry of the model has a length of 2 mm and the initial glass a height of 0.9 mm with a 0.1 mm layer of acid solution on top. The material point discretization was set such that the minimum distance between any pair of material points was 1 micron. It is worth noting here that the length scale in this model does not matter since the physics are self-similar and the results of this model will be analyzed in a unitless sense. The model is run dynamically to integrate the equations of motion and diffusive flow through time. The number of timesteps run is 50,000 with a time step increment of 74.72 ns.

## Supplementary Information

Supplementary Information.
