# Supplementary material for: A template-free, more environmentally friendly approach for glass micro-texturing
Source: Sci Rep. 2022 Jan 18;12:879. doi: 10.1038/s41598-022-04930-8 (PMC8766433; doi:10.1038/s41598-022-04930-8)

Supplementary information

Title: A template-free, more environmentally friendly approach for glass micro-texturing

Author information:

Order	Author Name<br>(First name Last name)	Affiliations	Contact Information	Country
1*+	Yuhui Jin	CRDC**	Jiny6@corning.com	United States
1+	Aize Li	CRDC**	Lia@corning.com	United States
3	Ross J Stewart	Corning Inc.	StewartRJ@corning.com	United States
4	Robert R Hancock	CRDC**	HancockRR@corning.com	United States
5	David E Baker	CRDC**	BakerDE@Corning.com	United States
6	Ruchirej Yongsunthon	CRDC**	YongsuntR@corning.com	United States
7	Kelleen K Hughes	CRDC**	HughesKK@corning.com	United States
8	David L Weidman	CRDC**	WeidmanDL@corning.com	United States

*: Corresponding author

+: Co- first author with equal contribution

**: CRDC: Corning Research and Development Corporation

Supplementary Table S1.

Glass	Acid	Additives	Treatment Temperature (°C)	Treatment Time (min)	Transmittance Haze (%)	Surface Roughness, Ra (nm)
A	20wt% citric acid	n/a	95	600	19.5	65.7
A	20wt% citric acid	n/a	95	810	38.8	94.8
A	20wt% citric acid	n/a	95	960	59	120.3
A	20wt% citric acid	n/a	95	300	0.3	
A	20wt% citric acid	n/a	95	360	0.6	
A	20wt% citric acid	n/a	95	420	3	
A	20wt% citric acid	n/a	95	480	8.6	
A	20wt% citric acid	n/a	95	300	0.1	
A	20wt% citric acid	n/a	95	360	0.8	
A	20wt% citric acid	n/a	95	420	1.4	
A	20wt% citric acid	n/a	95	480	7.9	
A	20wt% citric acid	n/a	95	510	7.9	
A	20wt% citric acid	n/a	95	540	12.8	
A	20wt% citric acid	n/a	95	570	16.6	
A	20wt% citric acid	n/a	95	600	21.5	
A	20wt% citric acid	1M $AlCl_3•6H_2O$	95	360	20.3	
A	20wt% citric acid	1M $CaCl_2•2H_2O$	95	360	5.1	
A	20wt% citric acid	1M $MgCl_2•6H_2O$	95	360	1.6	
A	20wt% citric acid	1M NaCl	95	360	0	
A	20wt% citric acid	1M $AlCl_3•6H_2O$	95	480	53.3	
A	20wt% citric acid	1M $CaCl_2•2H_2O$	95	480	18.4	
A	20wt% citric acid	1M $MgCl_2•6H_2O$	95	480	8.4	
A	20wt% citric acid	1M NaCl	95	480	0	
A	20wt% citric acid	1M $AlCl_3•6H_2O$	95	780	77.6	
A	20wt% citric acid	1M $CaCl_2•2H_2O$	95	780	59.4	
A	20wt% citric acid	1M $MgCl_2•6H_2O$	95	780	39.1	
A	20wt% citric acid	1M NaCl	95	780	2	
A	10wt% citric acid	n/a	96.5	420	3.7	
A	10wt% citric acid	n/a	96.5	480	6.3	
A	10wt% citric acid	n/a	96.5	660	21.7	
A	20wt% citric acid	n/a	97	360	10.4	
A	20wt% citric acid	n/a	97	420	17.3	
A	20wt% citric acid	n/a	97	480	27.9	
A	30wt% citric acid	n/a	97.3	240	7	
A	30wt% citric acid	n/a	97.3	360	35	
A	30wt% citric acid	n/a	97.3	480	55.2	

A	20wt% citric acid	1M FeCl3	98.7	65	1.2	17.8
A	20wt% citric acid	1M FeCl3	98.7	74	3.7	
A	20wt% citric acid	1M FeCl3	98.7	76	3.7	30.4
A	20wt% citric acid	1M FeCl3	98.7	88	11	43.8
A	20wt% citric acid	1M FeCl3	98.7	96	14.1	60.9
A	20wt% citric acid	1M FeCl3	98.7	115	34	87.8
A	20wt% citric acid	1M FeCl3	98.7	119	35.9	93.3
A	20wt% citric acid	1M FeCl3	98.7	146	68.2	136.5
A	20wt% lactic acid	n/a	95	960	50.4	
A	0.05M HCl	n/a	95	960	0	
A	20wt% malic acid	n/a	95	960	43	
A	20wt% citric acid	n/a	95	960	49	
A	20wt% tartaric acid	n/a	95	960	18.1	
A	20wt% ascorbic acid	n/a	95	960	0	
A	10M acetic acid	n/a	95	960	0	
A	20wt% citric acid	1M AlCl3•6H2O	100	60	0.7	
A	20wt% citric acid	1M AlCl3•6H2O	100	120	32.9	
A	20wt% citric acid	1M AlCl3•6H2O	100	180	94.6	
A	20wt% citric acid	1M AlCl3•6H2O	95	240	4	
A	20wt% citric acid	1M AlCl3•6H2O	95	480	80.6	
B	5wt% HCl	n/a	95	810	7.2	
B	5wt% HCl	n/a	95	1440	17.4	
B	5wt% HCl	n/a	95	2880	21.5	
C	20.4wt% HCl	n/a	104.7	360	0	
C	20.4wt% HCl	n/a	104.7	720	0.2	
C	20.4wt% HCl	n/a	104.7	1080	0.1	
A	20wt% citric acid	n/a	97	200	0.4	
A	20wt% citric acid	n/a	97	600	44.8	
A	10wt% citric acid	n/a	96.5	800	53.8	
A	20wt% citric acid	n/a	95	300	0.1	7.5
A	20wt% citric acid	n/a	95	360	0.7	14.2
A	20wt% citric acid	n/a	95	480	6.8	38.0
A	20wt% citric acid	n/a	95	600	19.8	63.6

Supplementary Figure S1. SIMS profile of the leaching layer depth and composition of glass A surface after 600-minute treatment in 20wt% citric acid at 95 °C. The depth profiles of silica and alumina suggests the treated glass A surface contains a 0.3µm (300nm) leaching layer with primarily silica and no alumina.

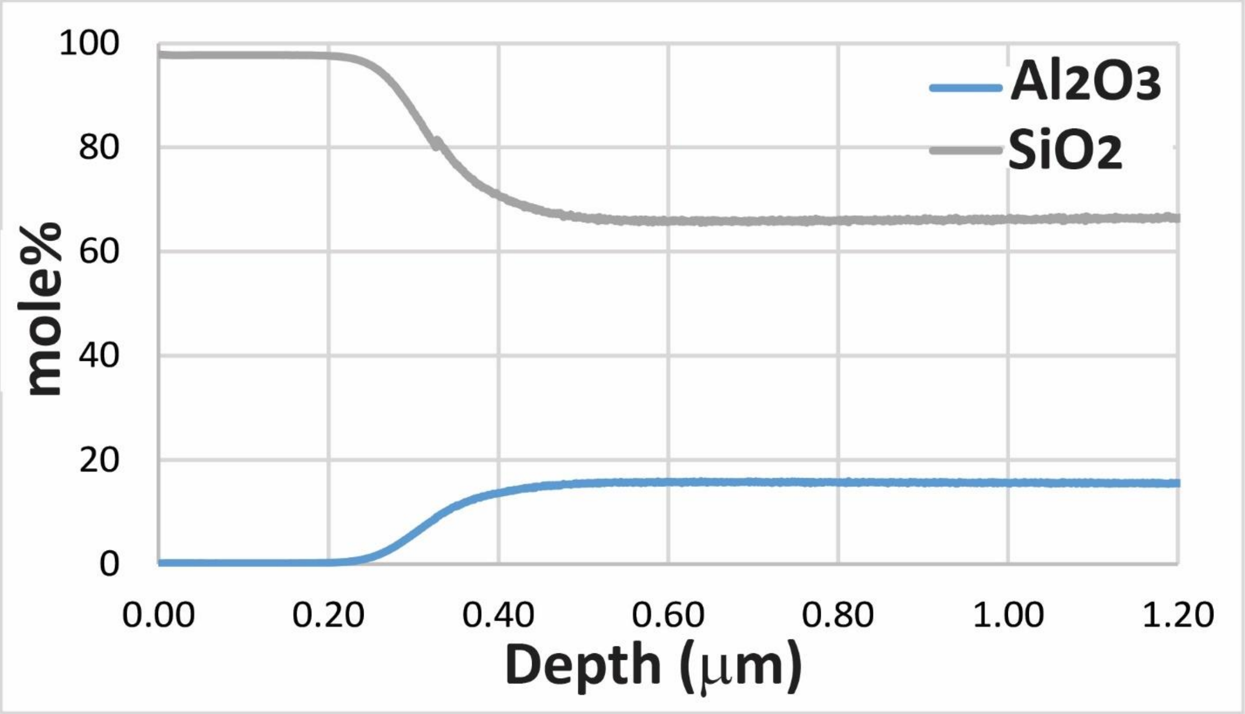

Supplement: Supplementary file 1 — Supplementary Information. [file 41598_2022_4930_MOESM1_ESM.pdf]
